# Neurotransmitter metabolites in milk ferments of *Leuconostoc mesenteroides* regulate temperature-sensitive heartbeats in an *ex ovo* model

**DOI:** 10.1016/j.heliyon.2024.e36129

**Published:** 2024-08-11

**Authors:** Mengke Zhang, Qing Chi, Mengru Lu, Jie Tang, Mingyu Zhang, Qianqian Wang, Deron R. Herr, Qing-Gao Zhang, Chun-Ming Huang

**Affiliations:** a Medical College of Dalian University, Dalian, 116622, China; b Health Medicine Translational Research Center, College of Dalian University, Dalian, 116622, China; c Sanford Burnham Prebys Medical Discovery Institute, La Jolla, CA, 92037, USA

**Keywords:** Ex ovo, Ferment, Heartbeat, *Leuconostoc mesenteroides*, Neurotransmitter

## Abstract

Accumulated evidence has supported the probiotic activity of *Leuconostoc mesenteroides* (*L. mesenteroides*) which can yield beneficial metabolites via fermentation. Here, bovine milk rich in phenylalanine（PHE) was used as a source for fermentation of *L. mesenteroides.* The complexes of PHE with bacterial phenylalanine hydroxylase (PheH) at two temperatures were revealed via molecular dynamics simulation. Two carbon hydrogen bonds and a Pi-Alkyl T-shaped interaction were newly formed at an active site of the PheH-PHE complex. The PheH interacted with two different hydrogen atoms in an amine of PHE via conventional hydrogen bonds at 37 °C, a temperature that accelerated the milk fermentation of *L. mesenteroides*. Twenty-eight metabolites including various neurotransmitters in fermented milk were identified and quantified by liquid chromatography coupled to quadrupole ion trap (Q-Trap) tandem mass spectrometry. *Ex ovo* injection of milk ferments into the yolk sac of chicken embryos enhanced a rising temperature-induced increase in heartbeats towards the normal resting level. The neurotransmitter-rich milk ferments hold potential for using to adjust energy metabolism, referred from heart rates, during fluctuating temperature conditions.

## 1 Introduction

*Leuconostoc mesenteroides* (*L. mesenteroides*) is a heterofermentative lactic acid probiotic bacterium which shows a high ability to decrease the formation of aldehyde, some of which are off-flavors in many ferments, foods transformed by the growth of metabolic activity of microbes [1], during fermentation. Dextransucrase of L. *mesenteroides* has been widely used to produce carbohydrates and derivatives such as dextran [2]. The bacterium displayed numerous beneficial effects, including antimicrobial activities against avian influenza (H9N2) virus [3] and oral *Porphyromonas gingivalis* [4] as well as anti-inflammatory properties for suppression of interleukin (IL)-12 and interferon (IFN)-γ production [5]. In addition, the anti-biofilm [6] and anti-allergy activities [7] of *L. mesenteroides* have been recently validated in mice. In the presence of different carbon sources which functioned as prebiotics, the probiotic *L. mesenteroides* yielded numerous beneficial metabolites such as short-chain fatty acids (SCFAs) [8]. One such SCFA produced by glucose fermentation, butyric acid, activated free fatty acid receptor 2 (Ffar2), efficiently increased insulin to ameliorate diabetes [9], and inhibited high fat diet (HFD)-induced abdominal fat in mice [8]. Electrons yielded by *L. mesenteroides* fermentation of linoleic acid remarkably suppressed the HFD-induced formation of 4-hydroxy-2-nonenal (4-HNE), a product of free radical-mediated lipid peroxidation [10]. Recently, two *L. mesenteroides* strains F-21 and F-22 have been isolated from human breast milk [11]. Results from genomic analysis indicated that these two strains exhibited immunomodulation capacity through interaction with Toll-like receptor (TLR) pathway components.

Both human and bovine milk are very abundant sources of amino acids including phenylalanine (PHE), tryptophan (TRP), glutamate (GLU), arginine (ARG), serine (SER) and tyrosine (TYR) [12]. PHE is an essential amino acid which humans are unable to sufficiently produce and need to obtain from diet. Lifelong dietary supplementation of PHE has also been recommended in patients with disorders affecting the nervous system such as phenylketonuria [13]. PHE is metabolized to TYR by phenylalanine hydroxylase (PheH) (EC1.14.16.1) [14,15]. TYR is subsequently converted to dihydroxyphenylalanine (L-DOPA) by tyrosine hydroxylase, a rate-limiting enzyme of biosynthesis of catecholamine neurotransmitters including dopamine (DO), epinephrine and norepinephrine (NE).

PheH with a single catalytic domain is monomeric in prokaryotes, but forms a tetramer in eukaryotes [16]. It has been reported that the activity of PheH in *Chromobacteria violaceum* (*C. violaceum*) bacteria was regulated by temperature, pH and metals [17]. The crystal structure of PheH has been analyzed and compared with that of PheH at 25 °C, the optimum growth temperature for *C. violaceum*, and 37 °C, the optimum core temperature for humans [18]. A thermodynamic analysis of PheH enzyme from the human pathogen *Legionella pneumophila* which grows well at temperature in the range 20–48 °C revealed high thermostability of PheH [19]. Fermentation of milk using various probiotic bacteria including *Lactobacillus bulgaricus* and *Streptococcus thermophilus* have created nutrition-dense foods which contain high content of minerals, vitamins, and essential fatty acids [20]. A bacteriocin has been produced by the milk fermentation of *L. mesenteroides* [21]. Both *Lactobacillus* spp. and *Bifidobacterium* spp. have been used for production of neurotransmitters such as γ-aminobutyric acid (GABA) during milk fermentation [22,23]. In this study, the interaction of PHE with *L. mesenteroides* PheH at two different temperatures (25 °C and 37 °C) was analyzed with the intention of understanding the optimal condition of PheH-PHE complex that may lead to sufficient production of various neurotransmitters during milk fermentation of *L. mesenteroides*.

It has been well documented that heart rate (HR) and energy expenditure were highly correlated [24]. HR is regulated continuously by activity of the autonomic nervous system and has been used as a surrogate for measurement of energy metabolism with changing human health [25,26]. Detection of HR can be achieved by non-invasively enumerating the heartbeats in chicken embryos [27], a model which allows to study the energy metabolism as a response of embryos to environmental insults [28]. Here, we injected the neurotransmitter-rich fermentation filtrates, named as milk ferments, of *L. mesenteroides* into chicken yolk sac and monitored changes in HR with fluctuating temperatures surrounding chicken embryos. Results in this study demonstrated for the first time that neurotransmitter-producing *L. mesenteroides* fermentatively metabolized bovine milk to yield neurotransmitters that significantly regulated temperature-induced HR in chicken embryos. The milk ferments of *L. mesenteroides* containing both inhibitory and excitatory neurotransmitters may provide a method for the regulation of energy metabolism.

## 2 Materials and methods

### 2.1 Molecular dynamics (MD) simulation of PheH-PHE complexes

A PHE bound crystal structure (identification number: 4JPY) of PheH from *C. violaceum* in protein data bank (PBD) was chosen for MD simulation. The Discovery Studio (DS) 2019 software was used for MD simulation, a tool for mimicking the interaction of PHE to PheH [29]. The macromolecule module was performed for PheH-PHE complex optimizations. The PheH-PHE complex was put into an orthorhombic box and solvated with an explicit periodic boundary solvation water model. To mimic the physiological environment, sodium chloride was added to the simulation system with an ionic strength of 0.145 [30]. A box of solvents containing 6125 water molecules were created. The Chemistry at HARvard Macromolecular Mechanics (CHARMM) force field [31] including parameters for bond stretching, angular distortions, interaction energy and atom-atom distances was uploaded onto MD simulation to analyze the PheH-PHE complexes at 25 °C and 37 °C, respectively. Total energy (kcal/mol) of interactions of Van Der Waals (VDW) and other electrostatic (ELEC) forces was calculated. The system temperature was set at 298.15 and 310.15 K within 4 ps. The MD simulation was started for 2 ns after 24 ps of equilibration. Lastly, the simulation snapshots were monitored for root mean square deviations (RMSD), a measurement of the average distance between atoms (alpha carbon) in PheH and PHE. The module of trajectory analysis was used to display the conventional or carbon hydrogen bonds as well as non-bonded (VDW, Pi-Pi T-shaped, Anide-Pi stacked and Pi-Alkyl) interactions between PheH and PHE. Frequency distribution of 500 different interactions of the conventional or carbon hydrogen bonds observed during the equilibrium of the MD simulation of PheH-PHE complexes at two different temperatures was presented.

### 2.2 Bacterial culture and fermentation

A *L. mesenteroides* strain originally isolated from curd cheeses was cultured in TSB (Sigma, St. Louis, MO, USA) overnight at 37 °C. After centrifugation at 5000 rpm for 10 min, bacteria were harvested in phosphate-buffered saline (PBS) for milk fermentation and other experiments. Conversion of optical density 600 nm (OD_600_) to colony forming units (CFUs) was performed to count bacterial numbers. For milk fermentation, the pure bovine milk (Xinle Dairy Industry Co., Ltd., Dalian, China) was added with or without *L. mesenteroides* bacteria (10^8^ CFU/mL) for further incubation at 25 °C or 37 °C. The formation of dense solids during milk fermentation was observed every 6 h.

### 2.3 Neurotransmitter metabolites in milk ferments

To prepare the freeze-dried powders, the fermented milk was frozen for 30 min at −20 °C and dried using the Eyela laboratory freeze-dryer under a vacuum pressure of 100 millitorrs for 12 h as previously described [32]. The freeze-dried powders of fermented milk (100 mg) were dissolved in 500 μL acetonitrile/water (1:1, v/v) solvent for analysis of liquid chromatography and mass spectrometry (LC-MS) using a Sciex 5500 quadrupole ion trap (Q-Trap) mass spectrometer (AB Sciex LLC, Framingham, MA, USA) equipped with an electrospray ionization (ESI) source. The mass spectrometer was connected to an Agilent 1290 infinity ultrahigh-performance liquid chromatography (UHPLC) system (Agilent Technologies, Inc., Santa Clara, CA, USA). Ammonium formate (25 mM) and acetonitrile in 0.1 % formic acid was used for mobile phase A and B, respectively. The source condition of ESI was set based on a published protocol [33]. The mode of multiple reaction monitoring (MRM) was performed to selectively quantify various neurotransmitter metabolites in fermented milk. A pair of ions (parent and daughter irons) of each individual metabolite in a mass spectrum were chosen for quantification. The Multiquant 3.0.2 software was utilized to extract chromatographic peak area and retention time. The standard curves using different concentrations of 28 neurotransmitter metabolites were established for quantification.

### 2.4 Ex ovo culture of chicken embryos

Egg shells of Hy-line brown eggs (Hy-line International, Iowa, USA) at day one of embryo development were opened with a drill (31.75 mm in diameter). The chick embryos were transferred into 50 mL Pyrex beakers covered with transparent plastic wraps and placed in an incubator at 38 °C with 70 % humidity. Four days after incubation, chick embryos in beakers were moved to a laminar flow cabinet at room temperature for 15 min. Right before immersion of the chick embryo-containing beaker in a 39 °C water bath, the yolk sac of embryo was injected with saline (50 μL) or supernatants collected from non-fermented or *L. mesenteroides*-fermented milk using a syringe with a 28 gauge (G) needle (Becton, Dickinson and Company, Franklin Lakes, NJ, USA). Supernatants collected from non-fermented or fermented milk were centrifuged at 5000 rpm for 10 min and subsequently filtered through 0.22 μm pore size filters (MilliporeSigma, Burlington, MA, USA) before injection into yolk sac. Temperature surrounding the chicken embryo (at the surface of transparent plastic wraps) was detected every min by an infrared thermometer (Shenzhen Jumaoyuan Technology Company, Shenzhen, China). Heartbeats of chick embryos at different temperatures were counted every min for 3, 15, and 15 min. All embryos were euthanized after experiments. The experiments were conducted in triplicate.

### 2.5 Statistical analysis

Data were analyzed by unpaired *t*-test using GraphPad Prism® 8.0 software. The *P*-values of <0.05 (*), <0.01 (**), and <0.001 (***) was considered statistically significant. The mean ± standard deviation (SD) was calculated from data collected from at least three independent experiments.

## 3 Results

### 3.1 Total interaction energy of PheH-PHE complex

**Fig. 1:**
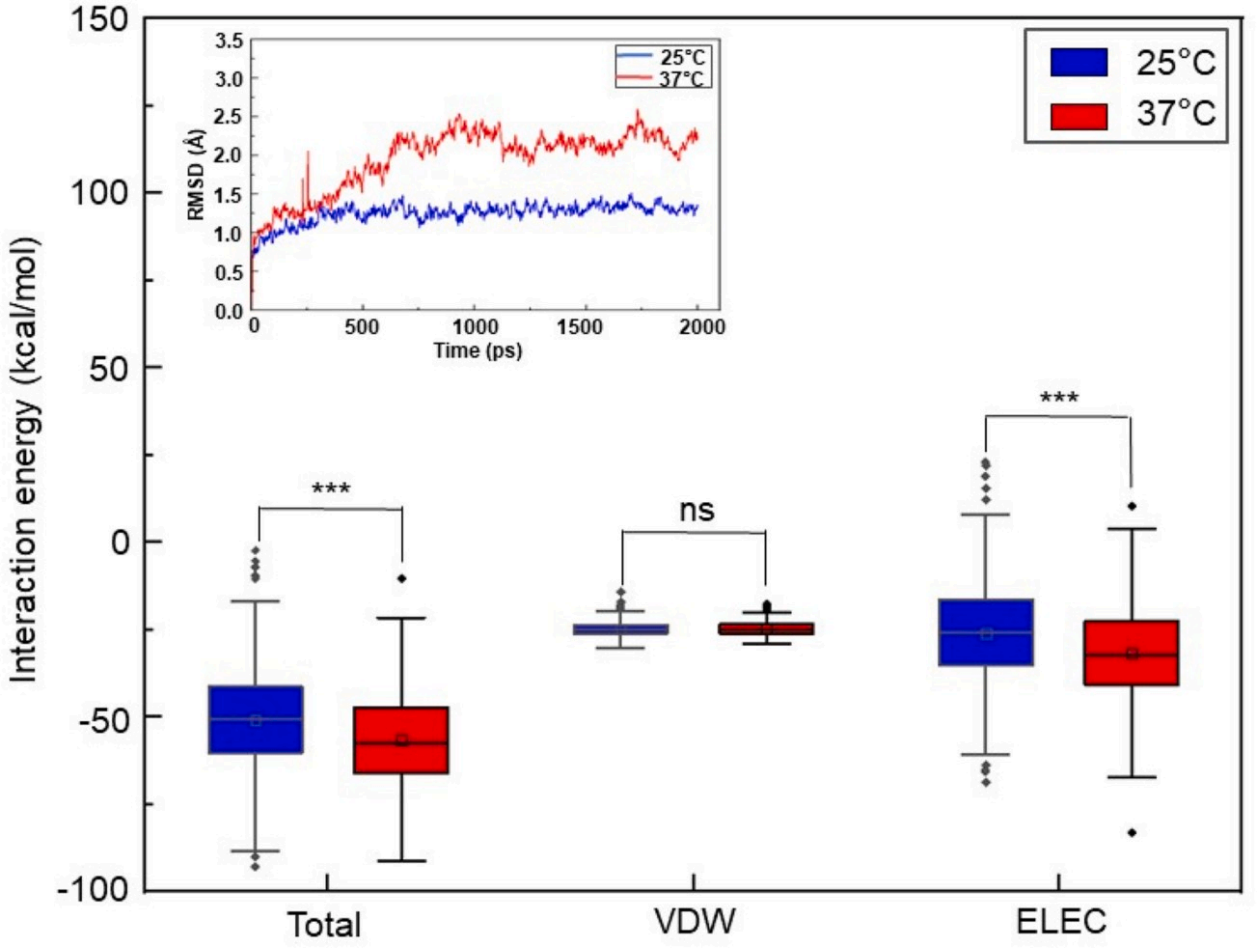
The MD simulation of PheH-PHE complex and calculation of energy of PheH and PHE interaction at two temperatures. The of PheH-PHE complex was analyzed with RMSD at 25 °C and 37 °C for 2000 ps (inserted panel). Total energy (kcal/mol) of interaction of PheH and PHE between 1000 and 2000 ps via VDW and ELEC was calculated. The *p*-value of <0.001 (***) from three different experiments with mean ± SD was shown. ns = non-significant.

The conformational changes undergone by PheH during the binding of PHE, an abundant amino acid in milk, at different temperatures may influence the efficiency of conversion of PHE to downstream metabolites. The MD simulation was performed to compare the different structural conformations of PheH-PHE complexes at 25 °C and 37 °C. Although the value of RMSD, an indicator of structural fluctuation, of PheH-PHE complex at 37 °C was higher than that of PheH-PHE complex at 25 °C, the RMSD values of PheH-PHE complexes at two temperatures became stable 500 ps after equilibrium (Fig. 1, inserted panel). Total energy of interaction of PheH and PHE between 1000 and 2000 ps at two temperatures was calculated. As shown in Fig. 1, the total energy of PheH-PHE complex at 25 °C was approximately −50 kcal/mol. The total energy at 37 °C was lower than that at 25 °C. Furthermore, compared to 25 °C, the temperature at 37 °C resulted in a significant decrease in the total energy of other electrostatic forces, but not VDW, in PheH-PHE complex. The data indicated that the interaction of PheH and PHE formed a relatively more stable complex at 37 °C as compared to that at 25 °C. Moreover, other electrostatic forces, not VDW, contributed to the reduction of interaction energy of PheH-PHE complex.

### 3.2 Forming of additional hydrogen bonds and a Pi-Alkyl T-shaped interaction in PheH-PHE complex

**Fig. 2:**
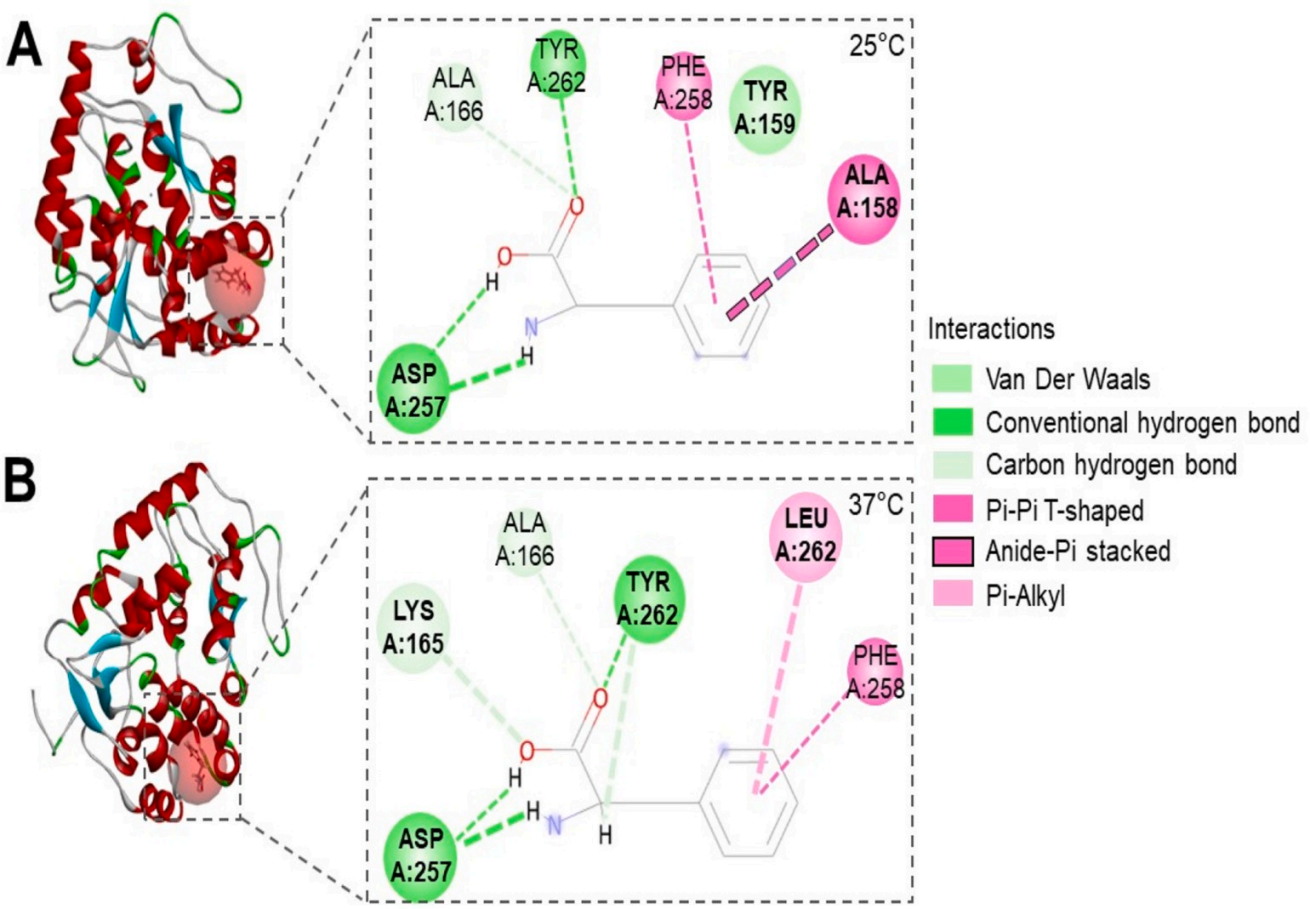
Interaction changes in the active sites of PheH-PHE complex. Structures of the active sites (dashed squares) of PheH-PHE complex at 25 °C (A) and 37 °C (B) were compared. Six amino acids at 25 °C [ASP, ALA (A:166 and A:158), TYR (A:262 and A:159), PHE] and six amino acids at 37 °C (ASP, LYS, ALA, TYR, LEU, PHE) interacted with PHE which was composed of a carboxylic acid (-COOH), an amine (-NH_2_), and a phenyl group (6 carbon atoms), via conventional (dark green) or carbon (light green) hydrogen bonds and non-bonded [VDW (mint green), Pi-Pi T-shaped (dark pink), Anide-Pi stacked (black framed pink) and Pi-Alkyl (light pink)] interactions.

A hydrogen bond is a main electrostatic force of attraction between a hydrogen atom and another electronegative atom [34]. Pi-stacking interaction is a noncovalent bond between the side chains of aromatic amino acids including PHE [35]. Thus, conventional or carbon hydrogen bonds as well as non-bonded (VDW, Pi-Pi T-shaped, Anide-Pi stacked and Pi-Alkyl) interactions in PheH-PHE complexes at 25 °C and 37 °C were examined. As shown in Fig. 2, two different structures of PheH-PHE complexes at 25 °C (Figs. 2A) and 37 °C (Fig. 2B) were exhibited. At both 25 °C and 37 °C, a conventional hydrogen bond was detected between TYR 262 in PheH and oxygen atom of a carboxylic acid (-COOH) of PHE. A carbon hydrogen bond was also present between alanine (ALA) 166 in PheH and a carboxylic acid (-COOH) of PHE in PheH-PHE complexes. Two additional interactions via carbon hydrogen bonds in PheH-PHE complexes occurred exclusively at 37 °C. The first carbon hydrogen bond was located between PheH TYR 262 and an amine (-NH_2_) of PHE. The second carbon hydrogen bond was present between lysine (LYS) 165 in PheH and oxygen atom of –COOH of PHE. Remarkably, aspartic acid (ASP) 257 in PheH interacted with two different hydrogen atoms in an amine (-NH_2_) of PHE via conventional hydrogen bonds. Besides hydrogen bonds, the noncovalent bonds between PheH and phenyl group (*aromatic ring of benzene*) *of PHE were characterized. PHE 258 in PheH reacted with phenyl group of PHE* via a Pi-*Pi T-shaped interaction at both 25°C and 37°C*. The interface of PHE with TYR 159 and ALA 158 in PheH through VMW and Anide-Pi stacked interaction, respectively, was only detectable at 37 °C. Furthermore, a Pi-Alkyl T-shaped interaction between leucine (LEU) 262 in PheH and PHE appeared at 37 °C, not 25 °C.

**Fig. 3:**
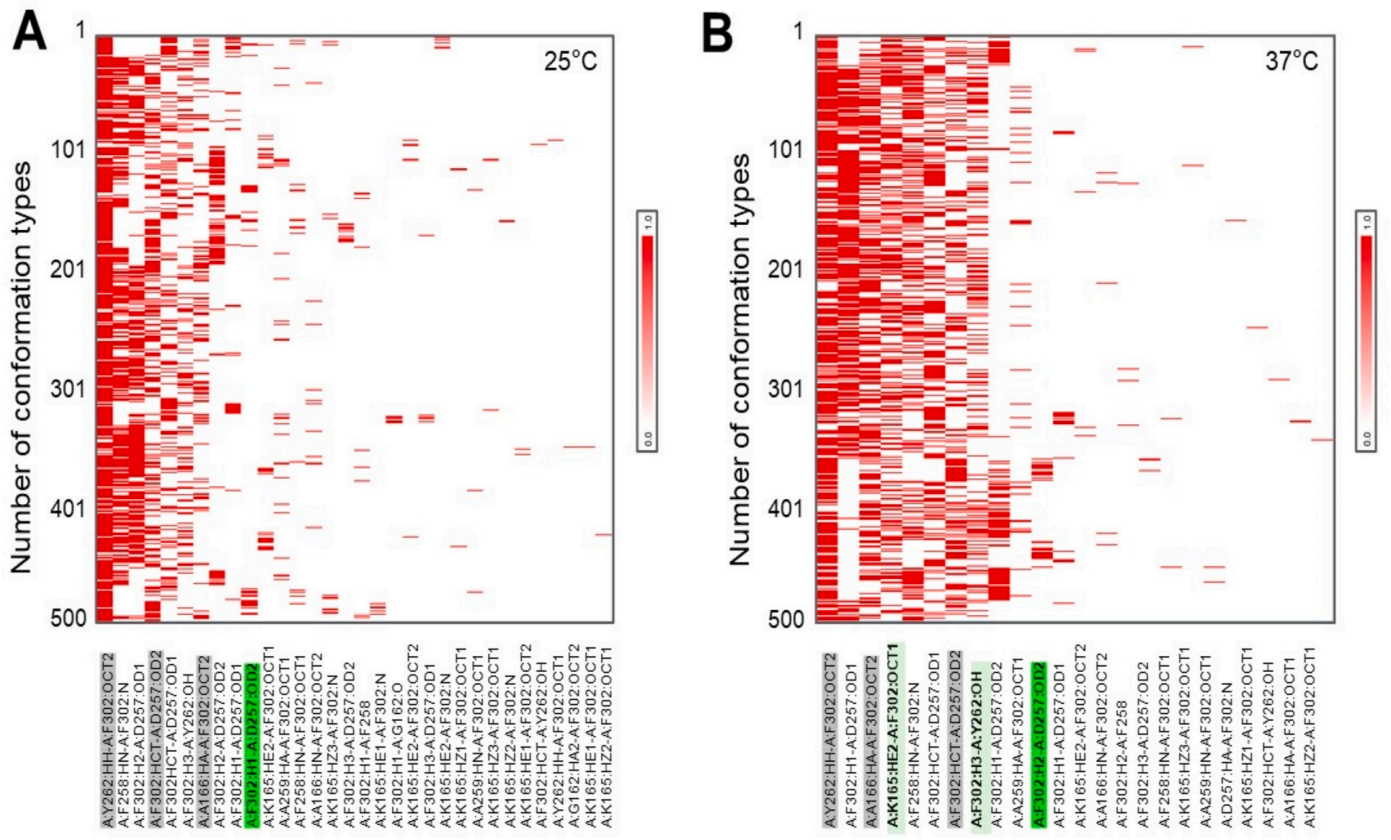
Frequency of various conformation of atom interactions via hydrogen bonds between PheH and PHE. The 32 and 24 conformations derived from interactions of atoms in PHE with atoms in different amino acids [ASP (D), ALA (A), TYR (Y), PHE (F), LYS (K), LEU (L)] in PheH at 25 °C (A) and 37 °C (B), respectively, are displayed (x-axis). *Frequency* distribution of 500 different conformation *in PheH-PHE complexes is shown (y-axis). Dark green indicates that ASP257 in PheH at* 25 °C (A:F302:H1-A:D257:OD2) and 37 °C (A:F302:H2-A:D257:OD2) *interacted with different H atoms of an amine in PHE. Light green indicates that two conformations (A:K165:HE2-A:F302:OCT1 and A:F302:H3-A:Y262:OH) exclusively were formed in PheH-PHE complexes at* 37 °C. Gray indicates that three conformations were commonly present in *PheH-PHE complexes at both* 25 °C and 37 °C.

Next, the analysis of frequency distribution of 500 different conformation types of PheH-PHE complexes via hydrogen bonds was conducted. As shown in Fig. 3, the temperature at 37 °C gave rise to an increase in the frequency of many different hydrogen bonding conformations of PheH-PHE complexes. In agreement with Fig. 2, *two conformations labeled as A:K165:HE2-A:F302:OCT1 and A:F302:H3-A:Y262:OH which were derived from the* carbon hydrogen bonding *of PheH LYS 165 and TYR 262 with* a carboxylic acid and an amine of PHE, respectively, were present frequently during the formation of PheH-PHE complex. Additionally, the conformations of PheH ASP 257 interacting with the first (A:F302:H1-A:D257:OD2) and second (A:F302:H2-A:D257:OD2) hydrogen atom of *an amine in PHE were often detected during the formation of* PheH-PHE complex *at* 25 °C (Figs. 3A) and 37 °C (Fig. 3B), respectively.

### 3.3 Synthesis of neurotransmitter metabolites in PHE-rich milk by L. mesenteroides fermentation

**Fig. 4:**
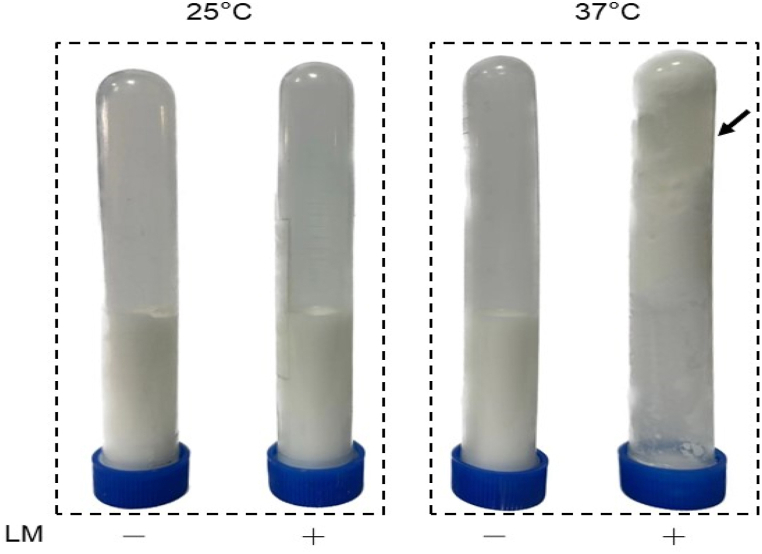
The formation of dense solids during milk fermentation of *L. mesenteroides*. Bovine milk with (+) or without (−) *L. mesenteroides* bacteria (10^8^ CFU/mL) was incubated at 25 °C or 37 °C. The formation of dense solids (arrow) in fermented milk was observed 42 h after adding *L. mesenteroides* into milk at 37 °C.

As shown in Fig. 4, there were no dense solids in milk without adding *L. mesenteroides* bacteria for incubation at either 25 °C or 37 °C. However, the dense solids were detected 42 h after adding bacteria and incubating at 37 °C, but not at 25 °C. The dense solids were later found in *L. mesenteroides*-added milk with a prolonged incubation time (96 h) at both 25 °C and 37 °C (Supplementary Material, Fig. S1). The result demonstrated that, compared to 25 °C, the 37 °C accelerated the milk fermentation of *L. mesenteroides.* It has been known that bovine milk contains high amounts of PHE, TRP, GLU, TYR and other amino acids which are the precursors for the synthesis of various neurotransmitters [36]. To determine if *L. mesenteroides* can fermentatively metabolize the amino acids in milk to neurotransmitters, the freeze-dried powders of fermented milk were dissolved in acetonitrile solvent and subjected to an UHPLC coupled Q-Trap mass spectrometer for identification and quantification of various amino acid metabolites.

**Fig. 5:**
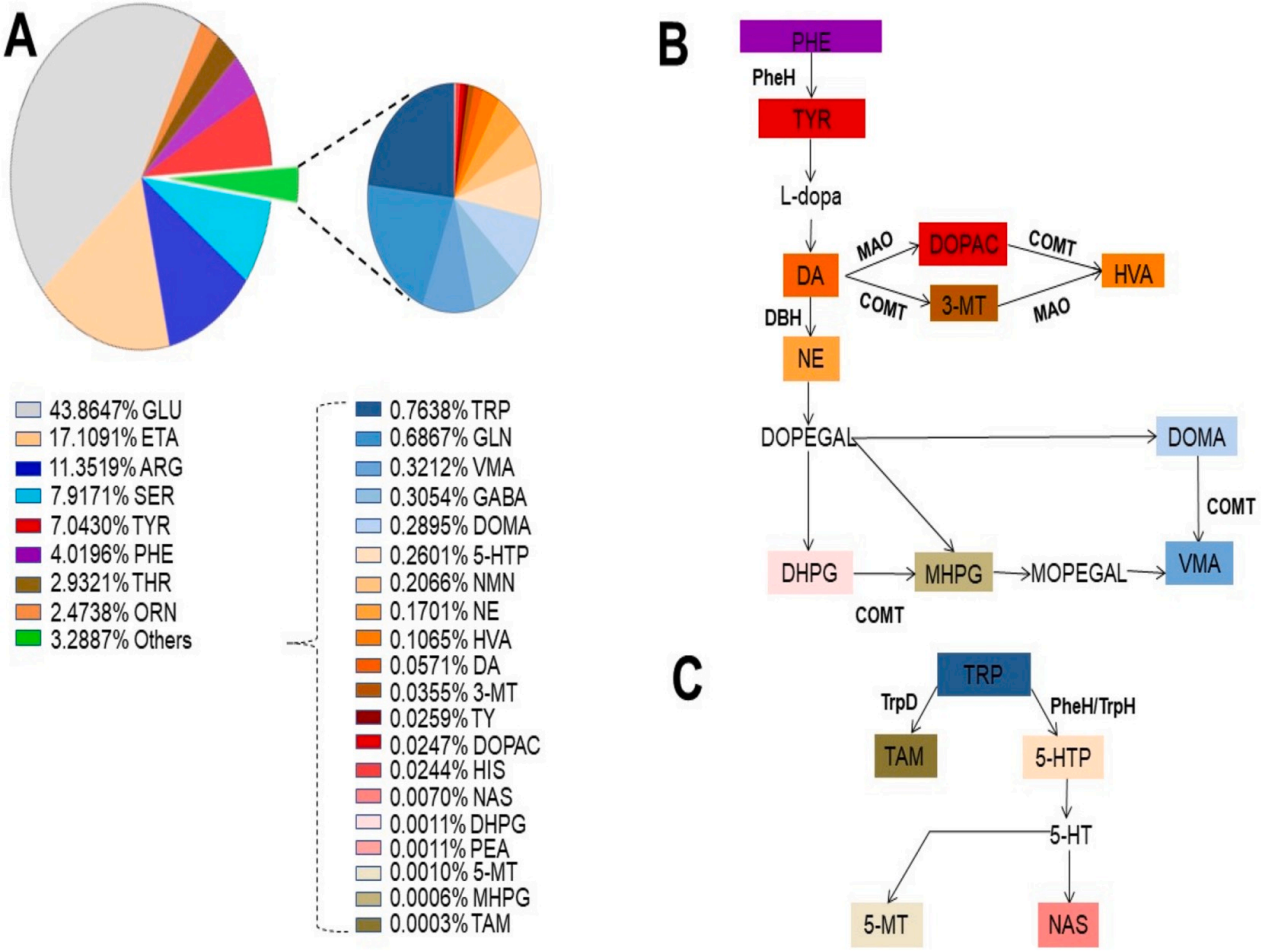
Neurotransmitter metabolites in milk ferments and their synthesis via the pathways of PHE and TRP metabolism. Neurotransmitter metabolites were identified and quantified (Supplementary Material, Table S1) by a UHPLC system coupled to a Sciex 5500 Q-Trap mass spectrometer. The percentages of eight metabolites including PHE with abundance greater than 2 % and twenty metabolites including TRP at relatively low abundance are displayed in two pie charts (A). Eleven and five detectable metabolites (color background) in milk ferments can be synthesized through the pathways of PHE (B) and TRP (C) metabolism, respectively. HIS, Histamine; NMN, Normetanephrine; PEA, *Phenylethylamine*; TY, Tyramine.

Twenty-eight metabolites including many known neurotransmitters were identified and quantified in Supplementary Material, Table S1. As shown in Fig. 5A, eight metabolites including GLU (43.9 %), the most abundant excitatory neurotransmitter in the vertebrate nervous system [37,38], ethanolamine (ETA) (17.1 %), ARG (11.4 %), SER (7.92 %), TYR (7.04 %), PHE (4.02 %), threonine (THR) (2.93 %) and ornithine (ORN) (2.47 %) existed in high abundance in fermented milk. Several neurotransmitters such as DO, an inhibitory or excitatory neurotransmitter [39], and NE, an excitatory neurotransmitter, derived from PHE metabolism (Fig. 5B) were detectable in fermented milk. The 3,4-dihydroxyphenylacetic acid (DOPAC), 3-Methoxytyramine (3-MT), and homovanillic acid (HVA) in fermented milk can be produced from DO degradation. The 3,4-dihydroxymandelic acid (DOMA), 3,4-Dihydroxyphenylglycol (DHPG), 3-Methoxy-4-hydroxyphenylglycol (MHPG), and vanillylmandelic acid (VMA) have known as metabolites of NE. Metabolites such as tryptamine (TAM), *5-hydroxytryptophan* (5-HTP), 5-Methoxytrytamine (5-MT), and N-acetylserotonin derived from TRP metabolism (Fig. 5C) were also present in fermented milk. The UHPLC spectra of HVA, NE and VMA were revealed in Supplementary Material, Fig. S2. Results above demonstrated that *L. mesenteroides* can ferment amino acids including PHE to generate various neurotransmitters.

### 3.4 Modulation of temperature-sensitive heartbeats by neurotransmitters in milk ferments

**Fig. 6:**
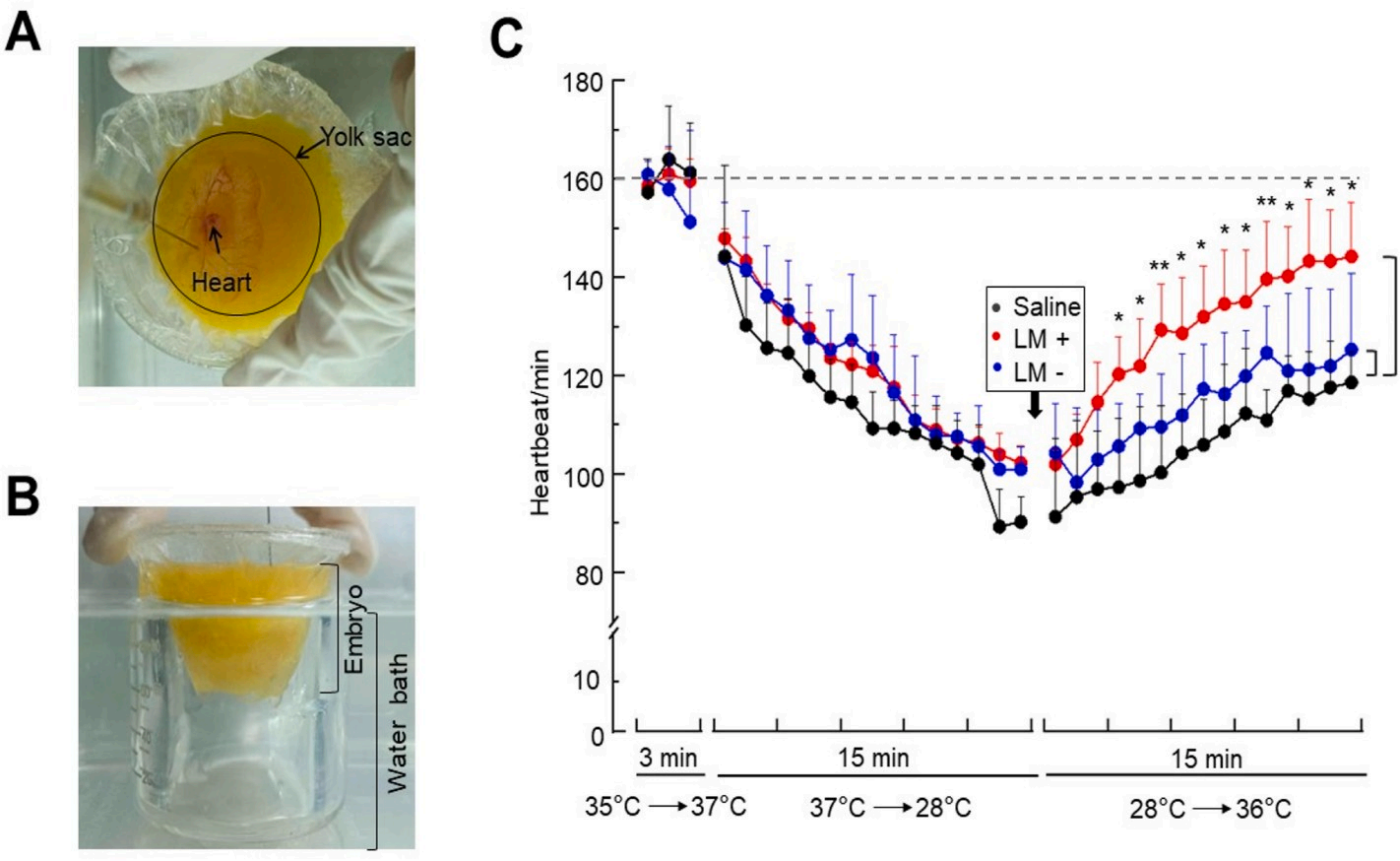
Regulation of heartbeats of chicken embryo HR by milk ferments. In an incubator, chick embryos in a Pyrex beaker (A) with surrounding temperature of 35–37 °C were moved to a laminar flow cabinet. The surrounding temperature of chick embryo at room temperature dropped from 37 °C to 28 °C in 15 min. Subsequently, the chick embryo in a Pyrex beaker was immersed to a water bath (B) to rise the ambient temperature from 28 °C to 36 °C in 15 min. Saline or supernatants collected from non-fermented (LM -) or *L. mesenteroides*-fermented (LM +) milk were injected into the yolk sac right before immersion into a water bath. Embryo HR was monitored continuously (C). The dashed line indicates normal resting HR at 160 average beats per min. Data are represented as mean ± SD with the *p*-values of <0.05 (*), and <0.01 (**) from experiments in triplicate. At least three chicken embryos per group were evaluated.

To understand if the *in vivo* supply of neurotransmitters in fermented milk affects the metabolic energy expenditure, the yolk sac of 5-day-old chicken embryo was injected with milk ferment or controls, and HR was monitored as a surrogate for energy metabolism. As an *ex ovo* model, the chicken embryo was transferred into a Pyrex beaker and exposed to different temperatures (Fig. 6A and B). The air temperature surrounding the chicken embryo was within a range of 35 °C–37 °C in an incubator, but fell into 28 °C in 15 min at room temperature. A marked decrease in HR was observed when embryos were moved to the room temperature from an incubator (Fig. 6C). The temperature surrounding the chicken embryo steadily increased to 36 °C when embryos were immersed into a water bath for 15 min (Fig. 6B). The average resting HR was 160 beats per min when embryos were placed in an incubator with a temperature of 35–37 °C. To investigate whether inoculation of neurotransmitter-rich milk ferments can enhance the effect of rising temperatures on the up-regulation of energy metabolism, the yolk sac of embryos was injected with filtrates of *L. mesenteroides* of milk ferments right before immersion in a water bath. The yolk sacs injected with saline or non-fermented milk filtrate served as controls. As shown in Fig. 6C, the immersion of chicken embryos led to a gradual increase in HR. In terms of rising temperature-induced HR increase, no significant difference existed when the yolk sac was injected with saline or milk filtrate. However, injection of milk ferments markedly enhanced HR of chicken embryo. After immersion of chicken embryo in a water bath for 15 min, the HR of milk ferment-injected embryos increased from 102 ± 3 to 144 ± 11 HR/min, reaching closer to the normal resting HR when embryos were in an incubator. The result suggested that inoculation of milk ferments *L. mesenteroides* increased energy metabolism against temperature-induced heartbeat fluctuation.

## 4 Discussion

Structural biology has allowed an understanding of the interaction of PheH with PHE at the atomic level. The RMSD value of PheH-PHE complex increased as the temperature was raised. However, the PheH-PHE complexes became stable after equilibrium for 500 ps. The PheH-PHE complex reached the most stable state with lower values of total and ELEC interaction energy when it was modeled at 37 °C (Fig. 1). PHE activated PheH via binding to the active site in the catalytic domain and/or a separate site in the N-terminal regulatory domain. Results from biochemical experiments revealed that mutation of ARG 270 in PheH efficiently diminished the binding of PHE to the active site [40]. Two mutations (TYR 198 and 204) of PheH were identified in phenylketonuria patients. Data from MD simulation predicted that these two mutations affect the activity of PheH [41]. Results from conformational analysis of mutated human PheH proteins by surface plasmon resonance (SPR) spectroscopy in real time have shown that substitution of TYR 138 in the active site influenced the activation of PheH by PHE [42]. Although the interactions of amino acids (ARG 270, TYR 138, 198 and 204) in PheH with PHE were not detectable in our analysis, we displayed two stable PheH-PHE complexes selected during 1000 to 2000 ps equilibrium. The different interactions of PHE in the active site of PheH were revealed from at 25 °C and 37 °C. Two carbon hydrogen bonds at LYS 165 and ALA 166 of PheH and a Pi-Alkyl T-shaped interaction at LEU 262 were newly formed in the active site of PheH-PHE complex at 37 °C. Furthermore, PheH formed conventional hydrogen bonds that joined ASP 257 to two different hydrogen atoms in an amine (-NH_2_) of PHE when temperature was switched from 25 °C to 37 °C (Fig. 2, Fig. 3).

Previous studies have demonstrated that the activation of PheH by PHE was primarily associated with changes in the tertiary/quaternary structure which can be affected by temperature [43,44]. The formation of dense solids in milk ferments of *L. mesenteroides* happened 54 h earlier when temperature was raised from 25 °C to 37 °C, suggesting that 37 °C may enhance the interaction of PHE with the active site of PheH (Fig. 4 and Fig. S1). It has been known that a regulatory domain of PheH, in addition to its active site, played a key role in activation by PHE [45]. Thus, a temperature which can promote the binding of PHE to both the active site and regulatory domain of PheH may be selected for acceleration of milk fermentation of *L. mesenteroides* in future experiments.

In this study, supernatants were collected from *L. mesenteroides*-fermented milk and filtered through 0.22 μm pore size filters to produce the bacteria-free fermentation filtrates, named as milk ferments, which were used for injection of chicken embryos (Fig. 6). The function of bacteria-free milk ferments with several neurotransmitters identified by UHPLC-Q-Trap mass spectrometry system was similar to that of postbiotics. By definition, postbiotics are products of probiotics and/or their metabolites that confer beneficial effects on the host [46]. It has been stated that the postbiotics of *L. mesenteroides* contain high amounts of SCFAs that exhibit antimicrobial activities [47]. GLU (43.87 %), the most abundant metabolite in milk ferments (Fig. 5A), is the precursor for the synthesis of glutamine (GLN), ARG, proline (PRO) and GABA. All GLN, ARG and GABA, but not PRO, were detectable in the milk ferments. It has been reported that the concentration of PRO in the chicken egg was insufficient to support embryonic growth during incubation [48]. Thus, injection of milk ferments with the high amount of GLU into the yolk sac of chicken embryo may increase the synthesis of PRO, promoting the embryonic development. Feeding mice with *Bifidobacterium adolescentis* probiotics converted GLN in milk to GABA [49]. The mouse model provided advantages of injecting live probiotic bacteria and measuring GLN metabolisms in different organs. Although injection of live probiotic bacteria into the yolk sac may lead to mortality of chicken embryo, the model of chicken embryo offers a suitable platform to dynamically monitor heartbeats without anaesthetization.

Our studies here demonstrate that postbiotics of *L. mesenteroides* from milk fermentation yield various neurotransmitters (Supplementary Material, Table S1) that regulate the heartbeats of chicken embryos. In addition to GLN, DO and NE, ARG was highly abundant representing 11.4 % of the neurotransmitters in milk ferments (Fig. 5). Notably, ARG is a known precursor for the synthesis of nitric oxide (NO), a neurotransmitter that controls blood pressure and improves circulation [45]. Although serotonin (5-HT) was untraceable in milk ferments, 5-HTP, an intermediate metabolite of TRP in the biosynthesis of 5-HT (Fig. 5C) was detected. Furthermore, 5-MT and NAS, two metabolites which can be formed from 5-HT, were present, despite their low abundance in milk ferments, suggesting that very little 5-HT may be produced during milk fermentation of *L. mesenteroides*. HVA composed of 0.107 % in milk ferments (Supplementary Material, Table S1) has been used as an indicator of dopaminergic activity [50,51]. Previous studies revealed that metabolic perturbation including HR changes are associated with plasma HVA levels in schizophrenia patients [52,53]. Interestingly, VMA, a norepinephrine end-metabolite, exhibited the activity of decreasing the HR in rats in a dose-dependent manner [54] although norepinephrine can increase HR. The milk ferments of *L. mesenteroides* with activities of regulating temperature-sensitive heartbeats in chicken embryos (Fig. 6C) contained both inhibitory and excitatory neurotransmitters. Future studies will include interfering neurotransmitter receptors in chicken embryos with specific blockers to investigate which neurotransmitters contributed to the regulatory effect of milk ferments of *L. mesenteroides* on HR.

The bacteria-free milk ferments were injected into the yolk sac of 5-day-old chicken embryo. The networks of blood vessels developed in the area vasculosa after 2 to 3 embryo incubation days (EID) [55]. Blood vessels in chicken embryos may transport blood along with products from injected milk ferments from the yolk sac to heart. It has been documented that, within 2–3 EID, chicken embryo underwent neurulation with beating hearts and complex nervous system [56]. Axons observed by electron microscopy was developed from 3 to 4 EID [57,58]. The central organs including circulatory system in chicken embryo with 11–12 EID became mature [59]. The *β-adrenergic* receptors possess high affinity binding of norepinephrine and a lower affinity binding of dopamine. It remains unclear whether the cardiac smooth muscle cells of 5-day-old chicken embryos express *β-adrenergic* receptors, although smooth muscle cells with *β-adrenergic* receptors surrounding a single layer of endothelial cells were found on the blood vessel wall of *chicken* chorioallantoic membrane after 5 EID [60]. Treatment of 6-day-old chicken embryos with NE caused a substantial increase in systolic and pulse pressure during heartbeats, indicating the presence of functioning *β-adrenergic* receptors [61]. Thus, it is worth using chicken embryos after 6 EID to verify the role of heart *β-adrenergic* receptors on regulation of temperature-sensitive heartbeats after injecting the milk ferments into the yolk sac.

## 5 Conclusion

MD simulation provided a reliable tool to unveil the atomic interaction of PheH with PHE in the active site, and determine an optimal temperature at 37 °C which resulted in the formation of a stable PheH-PHE complex and facilitated the milk fermentation of *L. mesenteroides*. Twenty-eight metabolites containing various neurotransmitters in milk ferments of *L. mesenteroides* were quantitatively analyzed by the UHPLC in conjunction with Q-Trap mass spectrometer. Lastly, a functional assay of milk ferments of *L. mesenteroides* was performed using an *ex ovo* model. Our results have shown for the first time that neurotransmitter-rich milk ferments regulated the heart rate, an indicator of energy metabolism, of chicken embryo.

## Funding

The study was supported by internal research funds of Dalian University and a grant from 10.13039/501100012166National Key Research and Development Program (2023YFC2508200).

## Data availability

Data associated with this study has not been deposited into a publicly available repository. All date will be included in this article, supplementary material, and references.

## CRediT authorship contribution statement

**Mengke Zhang:** Methodology, Investigation. **Qing Chi:** Methodology, Investigation, Conceptualization. **Mengru Lu:** Methodology, Investigation. **Jie Tang:** Investigation, Data curation, Conceptualization. **Mingyu Zhang:** Software, Methodology. **Qianqian Wang:** Software, Methodology, Conceptualization. **Deron R. Herr:** Writing – original draft, Data curation. **Qing-Gao Zhang:** Supervision, Project administration. **Chun-Ming Huang:** Writing – review & editing, Writing – original draft, Validation, Supervision, Resources, Methodology, Funding acquisition, Formal analysis, Conceptualization.

## Declaration of competing interest

The authors declare that they have no competing interests.
