# Supplementary material for: Neurotransmitter metabolites in milk ferments of Leuconostoc mesenteroides regulate temperature-sensitive heartbeats in an ex ovo model
Source: Heliyon. 2024 Aug 11;10(16):e36129. doi: 10.1016/j.heliyon.2024.e36129 (PMC11382174; doi:10.1016/j.heliyon.2024.e36129)

**Supplementary Material**

**Neurotransmitter metabolites in milk ferments of *Leuconostoc mesenteroides* regulate temperature-sensitive heartbeats in an *ex ovo* model**

Mengke Zhang^a¶^, Qing Chi^a¶^, Mengru Lu^a^, Jie Tang^a,b^, Mingyu Zhang^a,b^, Qianqian Wang^a,b^, Deron R. Herr^c^, Qing-Gao Zhang^a,b*^, Chun-Ming Huang^a,b*^

*^a^Medical College of Dalian University, Dalian, 116622, China*

*^b^Health Medicine Translational Research Center, College of Dalian University, Dalian, 116622, China*

*^c^Sanford Burnham Prebys Medical Discovery Institute, La Jolla, CA, 92037, USA*

Running title: Neurotransmitters in milk ferments regulate heartbeats in an *ex ovo* model

^¶^Equal contribution

^*^Corresponding authors.

*E-mail addresses*: zhangqinggao@dlu.edu.cn (QG. Zhang); huangjunming@dlu.edu.cn (CM. Huang)

*Keywords:* *Ex ovo*, Ferment, Heartbeat, *Leuconostoc mesenteroides*, Neurotransmitter

**Figure legends**

**Fig. S1.** The effect of prolonged incubation period on the formation of dense solids during milk fermentation. The dense solids (arrows) in fermented milk were found 96 h after adding *L. mesenteroides* (LM +) into milk at both 25°C and 37°C. No dense solids were formed in milk without (LM -) adding *L. mesenteroides*.

**Fig. S2.** The UHPLC spectra of three metabolites in milk ferments. HVA, NE and VMA involved in the pathway of PHE metabolism (Fig. 5B) were detectable in milk ferments of *L. mesenteroides*. The retention times of HVA, NE and VMA in the UHPLC spectra were 7.62, 9.97, and 6.82, respectively.

| Name | Retention time (min) | Ion pair (m/z) | Concentration (**μmol/L)** |
| --- | --- | --- | --- |
| GLU | 5.42 | 381.1/252.2 | 212.1362289 |
| ETA | 5.82 | 295.1/280.1 | 82.74340566 |
| ARG | 4.99 | 408.2/234.1 | 54.89954244 |
| SER | 5.34 | 338.9/324.0 | 38.28822283 |
| TYR | 8.84 | 648.2/340.2 | 34.06113871 |
| PHE | 7.38 | 399.1/157.1 | 19.43951954 |
| THR | 5.78 | 353.1/157.1 | 14.1797856 |
| ORN | 7.86 | 599.2/303.1 | 11.96377819 |
| TRP | 6.99 | 438.1/159.0 | 3.694000577 |
| GLN | 5.10 | 380.1/101.2 | 3.320657857 |
| VMA | 6.82 | 432.1/156.2 | 1.55325845 |
| GABA | 6.13 | 337.1/157.1 | 1.476865637 |
| DOMA | 8.57 | 651.1/170.1 | 1.400223992 |
| 5-HTP | 5.41 | 454.1/170.0 | 1.258021056 |
| NMN | 9.00 | 650.2/170.0 | 0.999270141 |
| NE | 9.97 | 869.2/851.0 | 0.822525136 |
| HVA | 7.62 | 416.1/171.1 | 0.51490503 |
| DA | 10.23 | 853.2/619.0 | 0.276120751 |
| 3-MT | 9.35 | 634.2/170.0 | 0.171432111 |
| TY | 9.49 | 604.2/170.0 | 0.125391904 |
| DOPAC | 9.08 | 635.2/401.0 | 0.119423989 |
| HIS | 8.98 | 578.2/170.0 | 0.118374576 |
| NAS | 7.41 | 452.1/170.1 | 0.033938653 |
| PEA | 8.28 | 354.9/157.1 | 0.005425814 |
| DHPG | 8.80 | 637.2/386.0 | 0.005286957 |
| 5-MT | 7.50 | 424.1/174.1 | 0.00492638 |
| MHPG | 7.11 | 418.1/171.0 | 0.00259465 |
| TAM | 7.85 | 394.2/130.0 | 0.001350674 |

**Table S1.** Retention times in UHPLC spectra, ion pair in LC-MS spectra, and concentrations of 28 metabolites in milk ferments of *L. mesenteroides*.

**Fig. S1.**


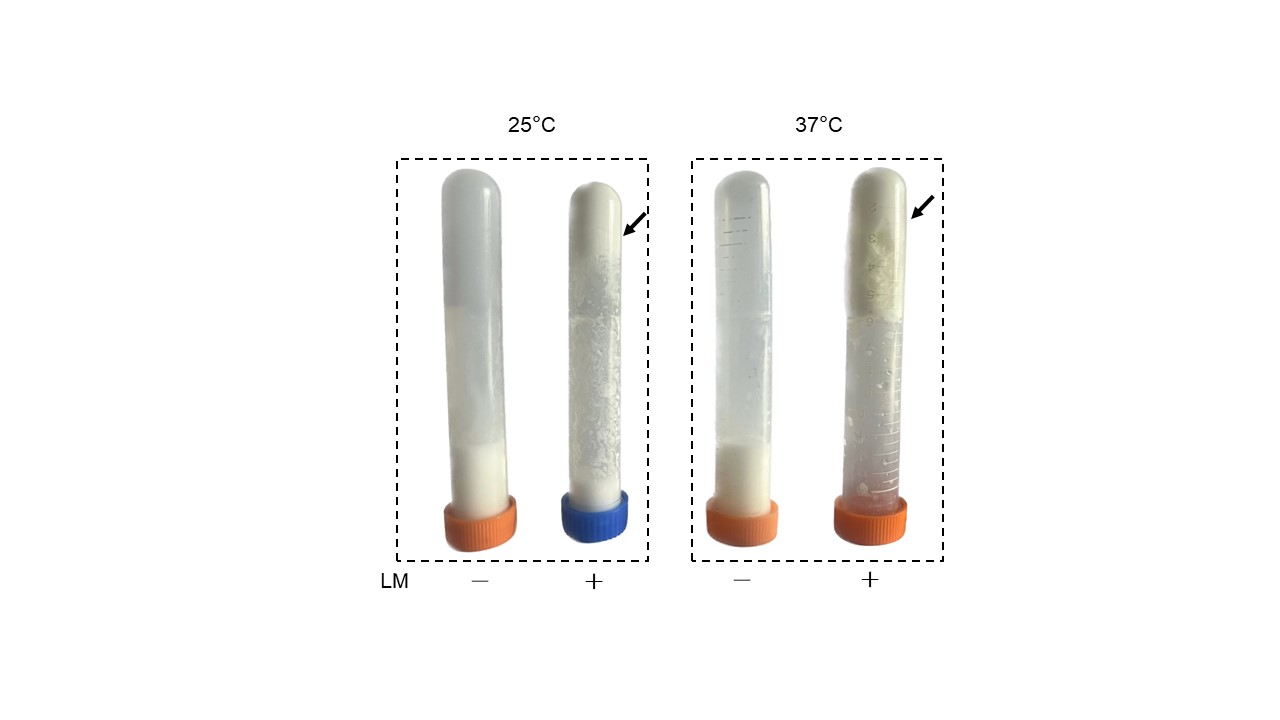


**Fig. S2.**


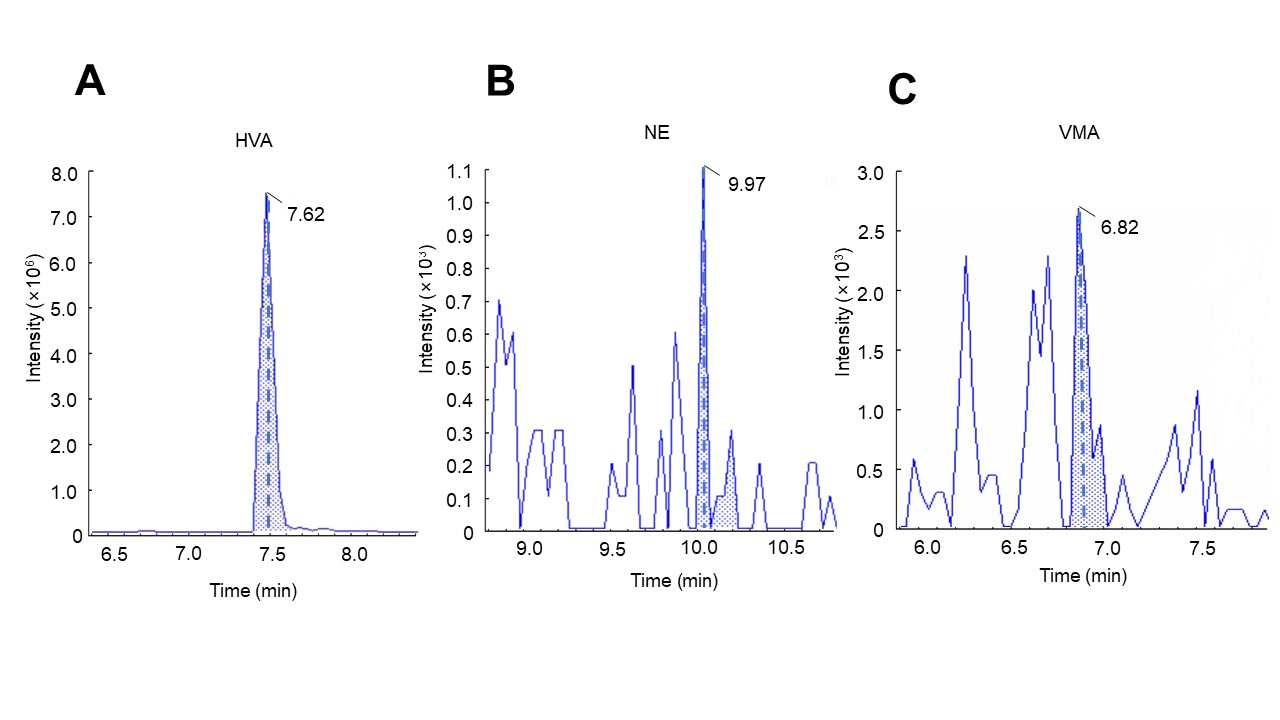

Supplement: Multimedia component 1 [file mmc1.docx]
